# RNA Interference of NADPH-Cytochrome P450 Reductase Results in Reduced Insecticide Resistance in the Bed Bug, *Cimex lectularius*

**DOI:** 10.1371/journal.pone.0031037

**Published:** 2012-02-07

**Authors:** Fang Zhu, Sarah Sams, Tim Moural, Kenneth F. Haynes, Michael F. Potter, Subba R. Palli

**Affiliations:** 1 Department of Entomology, College of Agriculture, University of Kentucky, Lexington, Kentucky, United States of America; 2 Division of Natural Sciences, Bluegrass Community & Technical College, Lexington, Kentucky, United States of America; Ghent University, Belgium

## Abstract

**Background:**

NADPH-cytochrome P450 reductase (CPR) plays a central role in cytochrome P450 action. The genes coding for P450s are not yet fully identified in the bed bug, *Cimex lectularius*. Hence, we decided to clone cDNA and knockdown the expression of the gene coding for CPR which is suggested to be required for the function of all P450s to determine whether or not P450s are involved in resistance of bed bugs to insecticides.

**Methodology/Principal Findings:**

The full length *Cimex lectularius* CPR (*ClCPR*) cDNA was isolated from a deltamethrin resistant bed bug population (CIN-1) using a combined PCR strategy. Bioinformatics and *in silico* modeling were employed to identify three conserved binding domains (FMN, FAD, NADP), a FAD binding motif, and the catalytic residues. The critical amino acids involved in FMN, FAD, NADP binding and their putative functions were also analyzed. No signal peptide but a membrane anchor domain with 21 amino acids which facilitates the localization of ClCPR on the endoplasmic reticulum was identified in ClCPR protein. Phylogenetic analysis showed that *ClCPR* is closer to the CPR from the body louse, *Pediculus humanus corporis* than to the CPRs from the other insect species studied. The *ClCPR* gene was ubiquitously expressed in all tissues tested but showed an increase in expression as immature stages develop into adults. We exploited the traumatic insemination mechanism of bed bugs to inject dsRNA and successfully knockdown the expression of the gene coding for ClCPR. Suppression of the *ClCPR* expression increased susceptibility to deltamethrin in resistant populations but not in the susceptible population of bed bugs.

**Conclusions/Significance:**

These data suggest that P450-mediated metabolic detoxification may serve as one of the resistance mechanisms in bed bugs.

## Introduction

During the past ten years, the bed bug has rapidly resurfaced throughout the world [1]–[3]. In an industry-wide survey of pest management firms in the United States, 76% considered bed bugs to be the most difficult pest to control [4]. It has been proposed that the global bed bug resurgence is partly due to the ubiquitous development of pyrethroid resistance [5]–[7]. Our previous studies determined a causal link between two identified knockdown resistance (*kdr*) mutations and deltamethrin resistance in bed bug populations, indicating decreased target-site sensitivity of voltage-gated sodium channels as one of the mechanisms of pyrethroid resistance. Interestingly, one population of bed bugs collected from Cincinnati (CIN-1) showed more than 12,765-fold deltamethrin resistance but no mutations were detected in the voltage-gated sodium channel gene [7]. Moreover, PBO, a P450 inhibitor, suppressed deltamethrin resistance in CIN-1 population [8]. These data suggest that cytochrome P450-mediated metabolic detoxification might be a principal mechanism responsible for deltamethrin resistance in some bed bug populations. However, the identity of P450s involved in detoxification of pyrethroids in bed bugs remains unknown.

Cytochrome P450s constitute one of the largest superfamilies of enzymes that play important roles in detoxification of xenobiotics [9], [10] as well as in biosynthesis and metabolism of endogenous compounds [11], [12]. The reaction of the P450 system requires electrons transferred from Nicotinamide Adenine Dinucleotide Phosphate (NADPH) to the P450 heme center by a Cytochrome P450 partner enzyme, NADPH-Cytochrome P450 Reductase (CPR) [13]. Although, multiple P450 genes have been found in the genomes of insects (http://drnelson.uthsc.edu/cytochromeP450.html), typically only one *CPR* gene exists in each insect genome. CPR is a multidomain protein which belongs to the electron transfer flavoproteins family [14] containing both Flavin Adenine Dinucleotide (FAD) and Flavin Mononucleotide (FMN) domains [15]. In addition to cytochrome P450s, CPR also serves as the electron donor protein for several oxygenase enzymes found in the endoplasmic reticulum of most eukaryotic cells [16]–[19]. Genes coding for *CPR*s have been identified and characterized from a few species of insects, such as house fly, *Musca domestica* L. [20]–[22], fruit fly, *Drosophila melanogaster* (Meigen) [23], silkworm, *Bombyx mori* L. [24], cabbage armyworm, *Mamestra brassicae* (L.) [25], mosquitoes, *Anopheles gambiae* Giles [26] and *Anopheles minimus* Theobald [27]. Sequences of *CPR* cDNAs are also available for many other insect species (Table S1).

With critical biological function associated with cytochrome P450s, insect CPRs have been placed in a vital path in metabolism-based insecticide resistance and were considered as the novel target for the development of synergists [26], [28]. In the current study, the *Cimex lectularius* CPR (ClCPR) cDNA was cloned and the gene coding for CPR was silenced in both deltamethrin resistant and susceptible populations of bed bugs. The data collected helped to reveal the role of P450-mediated metabolic detoxification in the deltamethrin resistance of bed bugs.

## Materials and Methods

### The bed bugs

Three bed bug colonies were used in this study. One insecticide-susceptible colony, LA-1, collected in 2006 in Los Angeles, CA [29] was maintained in the laboratory without any insecticide exposure. Two deltamethrin resistant populations of bed bugs, CIN-1 (Cincinnati, OH, contains no *kdr* mutations) and NY-1 (Plainview, NY, contains two *kdr* mutations) were collected from human dwellings in the United States during 2006–2008 [9] and maintained in the laboratory by using a parafilm-membrane feeder. Bed bugs were kept in screened containers and fed with 37°C heparinized chicken blood or rabbit blood with sodium citrate through a thinly stretched parafilm membrane [30]. Blood was purchased from Hema Resource and Supply Company (Aurora, OR). Bed bugs were reared at 27°C, 65±5% RH, and a photoperiod of 14∶10 (L∶D) h.

### RNA extraction, cDNA preparation and Cloning

Total RNA was isolated from 3 CIN-1 adults using the TRI reagent (Molecular Research Center Inc., Cincinnati, OH) and the RNA was treated with DNase I (Ambion Inc., Austin, TX). cDNA was synthesized using iScript cDNA synthesis kit (Bio-Rad Laboratories, Hercules, CA) with DNase I treated total RNA as a template. The PCR products were amplified using primer pair NADPHF/NADPHR (Table S2) that was designed based on a conserved amino acid region found in 10 insect *CPR* sequences. The PCR products were cloned into pGEM®-T Easy Vector Systems (Promega) and sequenced. Cloning and sequence analyses of P450 gene fragments were repeated at least three times with different preparations of RNAs. Three clones from each replication were sequenced.

### Rapid amplification of cDNA ends (RACE) of the putative *ClCPR* gene fragment

RACE was carried out using the SMARTer^Tm^ RACE cDNA Amplification Kit (Clontech) as described in the manufacturer's manual. The first strand cDNAs were synthesized with SMARTScribe™ Reverse Transcriptase using CIN-1 RNA as a template. The double-stranded cDNA was synthesized following the protocol described in the manufacturer's manual (Clontech). The 5′ and/or 3′ ends of the P450 cDNA fragments were amplified by PCR using adapter primers UPM and NUP and gene specific primers generated based on the 5′ and/or 3′ end sequences of the putative *ClCPR* transcript (Table S2). The full length of putative *ClCPR* cDNA was subsequently generated by RT-PCR using specific primer pair of ClCPRF/ClCPRR (Table S2) synthesized based on the 5′and 3′end sequences of the putative *ClCPR* mRNA. Cloning and sequence analyses of the *ClCPR* transcript were repeated at least three times, and three clones from each replicate were verified by sequencing.

### *In silico* structural analysis

The Isoelectric point (pI) and Molecular Weight (MW) of ClCPR were calculated by an ExPASy proteomics tool, Compute pI/Mw (http://web.expasy.org/compute_pi) from the Swiss Institute of Bioinformatics. The signal peptide and protein subcellular localization of ClCPR were analyzed at the SignalP 3.0 server (http:www.cbs.dtu.dk/services/SignalP/) and WoLF PSORT (http://wolfpsort.org/). The secondary structure, binding domains, and catalytic residues were predicted by PHYRE2 Protein Fold Recognition Server (http://www.sbg.bio.ic.ac.uk/phyre2/html/), Pfam 25.0 (2011, http://pfam.sanger.ac.uk/), and a conserved domain search on the NCBI website (http://www.ncbi.nlm.nih.gov/Structure/cdd/cdd.shtml) [31]. The protein tertiary structure of ClCPR was predicted by using the I-TASSER server (http://zhanglab.ccmb.med.umich.edu/I-TASSER/) [32] and then the PDB coordinate file of the highest ranking model was loaded into Chimera (http://plato.cgl.ucsf.edu/chimera/docs/credits.html) for molecular visualization and modification. The transmembrane helices of ClCPR were analyzed by TMHMM Server 2.0 (htt://www.cbs.dtu.dk/services/TMHMM-2.0/). The hydrophobicity of ClCPR was predicted by an on line molecular tool, Protein Hydrophobicity Plots (http://www.vivo.colostate.edu/molkit/hydropathy/).

### Phylogenetic tree construction

All *CPR* sequences in insects which have the complete open reading frames (ORFs) were extracted from the National Center for Biotechnology Information (NCBI) (Bethesda, MD) (http://www.ncbi.nlm.nih.gov/). The insect CPR amino acid sequences were analyzed using ClustalW alignment through Molecular Evolutionary Genetic Analysis software version 5 (MEGA 5) (http://www.megasoftware.net/) [33]. To improve the alignments, the pair wise alignment was performed with the gap opening penalty at 10, and the gap extension penalty left at default 0.1. The multiple alignments were conducted with the gap opening penalty at 3 and the gap extension penalty at 1.8 [34]. The sites containing missing data or alignment gaps were eliminated in a pair-wise manner. A p-distance<0.8 when carrying out the compute overall mean distance suggested the alignment was acceptable [34]. Subsequently, the alignment result was converted to a MEGA file (.meg) and submitted to construct the phylogenetic tree with neighbor-joining algorithm. A total of 2,000 bootstrap replications were used to test of phylogeny. Ultimately, the selected tree was created with cut-off value of 50%.

### Quantitative real time PCR (qRT-PCR) and reference gene selection

qRT-PCR was performed in MyiQ single color real-time PCR detection system (Bio-Rad Laboratories, Hercules, CA). Total RNA was isolated from 3 female bed bugs at 5 days after RNAi treatment using the TRI reagent (Molecular Research Center Inc., Cincinnati, OH). The RNA was treated with DNase I (Ambion Inc., Austin, TX). cDNA was synthesized using iScript cDNA synthesis kit (Bio-Rad Laboratories, Hercules, CA). DNase I treated total RNA was used as a template. Each qRT-PCR reaction (10 µl final volume) contained 5 µl FastStart SYBR Green Master (Roche Diagnostics, Indianapolis, IN), 1.2 µl of cDNA, and 0.6 µl each of forward and reverse gene specific primers (Table S2, stock 10 µM). An initial incubation of 95°C for 3 min, followed by 40 cycles of 95°C for 10 s, 55°C for 20 s, and 72°C for 30 s settings were used. A fluorescence reading determined the extension of amplification at the end of each cycle. Each experiment was repeated at least three times using independent biological samples.

The suitability of four reference/control genes, *rpl11*, *rpl8*, *rps16* and *hsp70* was evaluated with the *Bestkeeper* software package [35], [36]. This program was used not only to calculate potential reference genes, but also to assess the effects of RNAi on target genes. We designed primers for reference genes based on the EST sequences in the GenBank database (GenBank Accession Nos.: *rpl11*, EZ419774; *rpl8*, EZ419796; *rps16*, EZ419784; *hsp70*, EZ419756). Primers used for amplification reference genes are shown in Table S2. Relative expression levels for specific genes, in relation to the most reliable reference gene, were calculated by the 2^−ΔΔCT^ method [37].

### dsRNA injection by traumatic insemination mimicking

The dsRNA was synthesized using the MEGscript RNAi Kit (Ambion Inc., Austin, TX). Genomic DNA was isolated from CIN-1 adults using DNeasy Tissue Kit (QIAGEN). Genomic DNA and T7 promoter-containing PCR primers (Table S2, with T7 RNA promoter sequence (TAATACGACTCACTATAGGG) appended at the 5′ ends of both sense and antisense specific primers) were used in a PCR reaction to obtain gene specific fragments containing T7 promoter sequence on both ends. PCR product (200–400 bp) was used as a template to synthesize dsRNA. The same length PCR fragments were obtained using either genomic DNA or cDNA as a template suggesting that there is no intron in the region of this gene used for dsRNA preparation. For the dsRNA purification, phenol/chloroform extraction followed by ethanol precipitation method was applied. dsRNA was diluted in nuclease-free water to 4–5 µg/µl for injection into bed bug adults. The one week old female adults were anaesthetized with ether vapor for 10 min and placed on a glass slide covered with double-sided tape. The dsRNA (∼1.25 µg) was injected into the spermalege of the abdomen with an injection needle pulled out from a glass capillary tube using a needle puller (Idaho Technology, Salt Lake City, Utah). The spermalege is where the cuticle of the female is punctured during traumatic insemination.. Prior to injection, the glass needles were sterilizeed by soaking in 100% ethanol for 12 h. Controls were injected with the dsRNA using bacterial *malE* gene as a template. After injection, insects were removed from the glass slide, allowed to recover for 3 h at room temperature, then returned to normal rearing conditions.

### Bioassays with deltamethrin after dsRNA injection

In the preliminary studies, bed bug adults were treated with serial dilutions of technical grade deltamethrin (99% active ingredient, Bayer Environmental Science, St. Louis, MO) prepared in acetone. A discriminating dose (causing approximately 50% of mortality) of deltamethrin was applied for the bioassays. Acetone was used as a control. The solution was dropped on the thorax of the bugs (1 µl/drop) using a PB-600 repeating dispenser (Hamilton Co., Reno). The mortality was determined at 24 h after treatment. Mean and standard errors for each time point were obtained from at least three independent bioassays.

### Statistical analysis

Statistical analyses were carried out using SAS software (v9.1, SAS Institute Inc., Cary, NC). Student's *t*-test (two-tailed paired *t*-test) was used to compare the gene expression and mortality difference between two samples. The differences among samples were analyzed by One-way ANOVA, followed by Duncan multiple mean separation techniques. The level of significance was set at *P*<0.05.

## Results

### Cloning, sequence analysis, and structural modeling of ClCPR

The overall strategy of cloning the full length of ClCPR is shown in Figure S1. Briefly, a partial putative *ClCPR* cDNA fragment was amplified from deltamethrin resistant population, CIN-1, by multiple PCR amplifications using degenerate primers, NADPHF and NADPHR designed based on CPR sequences identified in other insect species (Table S2). BLAST analysis of the amino acid sequence predicted from the partial putative *ClCPR* cDNA sequence showed that the sequence encoded ClCPR and shared 81% amino acid similarity with the *CPR* sequence from the body louse, *Pediculus humanus corporis*. To amplify the 5′ and 3′ ends of this gene, 5′-RACE and 3′-RACE reactions were conducted using the adapter primers and gene specific primers designed based on the 5′ and 3′ end sequence of the putative *ClCPR* cDNA fragment, respectively (Table S2). The sequences of the 5′-RACE and 3′-RACE fragments overlapped with the *ClCPR* cDNA fragment sequence, identifying them as the 5′ and 3′ ends of the putative *ClCPR* gene. Subsequently, the full length *ClCPR* cDNA was amplified from CIN-1 bed bugs using PCR and the specific primers (ClCPRF and ClCPRR) designed based on the 5′ and 3′ end sequences of the putative *ClCPR* gene (Fig. S1, Table S2). The ClCPR amino acid sequence was aligned with the amino acid sequences of other CPRs from taxonomically diverse insect species. The sequence of ClCPR showed 65%, 68%, 66%, and 70% amino acid identity respectively with the CPR sequences of *An. gambiae*, *D. melanogaster*, *M. brassicae*, and *M. domestica*, (Table S1).

**Figure 1:**
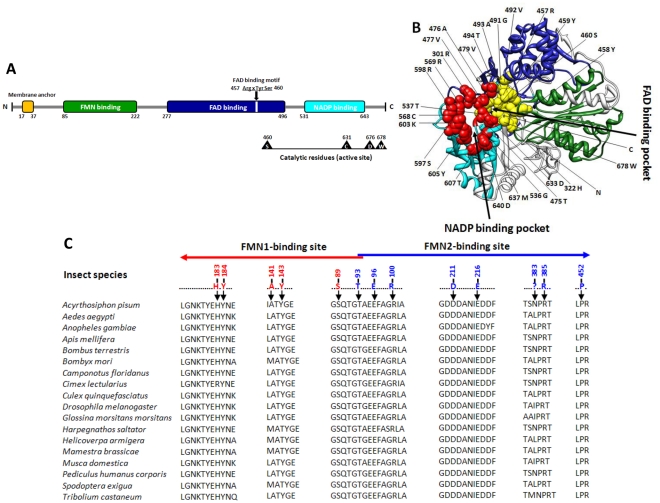
Structure of ClCPR. (A) Schematic drawing of ClCPR with membrane anchor (orange bar), conserved binding domains (green bar-Flavodoxin, blue bar-FAD binding, cyan bar-NADP binding), FAD binding motif (ArgxTyrSer), and catalytic residues (Ser-Cys-Asp-Trp). (B) Predicted three-dimensional structure of ClCPR with emphasis on FAD and NADP binding pockets. Three binding domains are highlighted in different colors (green-Falvodoxin, blue-FAD binding, and cyan-NADP binding) in the model. Fifteen amino acids composing the NADP binding pocket are highlighted as red spheres. Thirteen amino acids which constitute the FAD binding pocket are highlighted as yellow spheres. N- and C- termini are also labeled in the ClCPR tertiary structure. (C) Sequence alignment for FMN binding sites in insect *CPR*s. Residues constituting the FMN1-binding site were labeled with red numbers, and the residues constituting the FMN2-binding site are labeled with blue numbers. The arrows show the direction from the N terminus to the C terminus. All insect CPR amino acid sequences were extracted from NCBI (Bethesda, MD) (http://www.ncbi.nlm.nih.gov/).The sequence alignment was performed using ClustalW through MEGA 5 [33]. The cDNA sequence of ClCPR has been deposited in the GenBank database, accession number, JQ178363.

The *ClCPR* cDNA sequence contained an ORF of 2037 nucleotides encoding 679 amino acids. The pI and Mw were predicted as 5.55 and 77.01 kilodaltons, respectively. No signal peptide was found within ClCPR, but the membrane anchor which facilitates the localization of ClCPR on the endoplasmic reticulum was identified (Fig. 1A). All functional domains involved in the binding of cofactors, FMN, FAD and NADPH were identified in the predicted ClCPR protein primary and tertiary structures (Fig. 1). Three amino acid residues including Arginine 457, Tyrosine 459, and Serine 460 constitute a FAD binding motif which is similar to the conserved FAD binding domain [38] (Fig. 1A). The ClCPR catalytic residues (active site) comprise Serine 460, Cysteine 631, Aspartic 676, and Tryptophan 678 (Fig. 1A). These residues have been shown to be critical in the hydride transfer reaction catalyzed by rat CYP Oxidoreductase [39], [40].

To understand the structure which may direct function, the three-dimensional model of ClCPR was predicted. As shown in Fig. 1B, ClCPR consists of three distinct protein binding domains, FMN binding domain, FAD binding domain, and NADP binding domain. The FAD and NADP binding domains present at the C terminus are similar to the FAD/NADP domain reported in human CPR [41]. The FAD binding pocket is composed of thirteen amino acids, and the NADP binding pocket is composed of fifteen amino acids (Fig. 1B). At the N terminus, the FMN domain with two FMN binding sites is structurally similar to flavodoxins and interacts with the redox-partner binding site of the P450s [42] (Fig. 1C). During a catalytic cycle, ClCPR is predicted to transfer a hydride ion derived from NADPH to FAD, and then FAD transfers electrons to FMN, from where they are delivered to acceptor proteins (such as P450s) [15], [43].

### Subcellular localization of ClCPR

No conserved signal peptide was identified at the N-terminal end of ClCPR suggesting that ClCPR is retained in the cytoplasm. The CPR is anchored on the membrane of endoplasmic reticulum by an N-terminal hydrophobic segment [44]. A deduced hydrophobic transmembrane region consisting of 21 amino acids identified at the N-terminal end of ClCPR may be involved in the membrane anchor function (Fig. S2).

### Phylogenetic relationship of ClCPR with other insect CPRs

**Figure 2:**
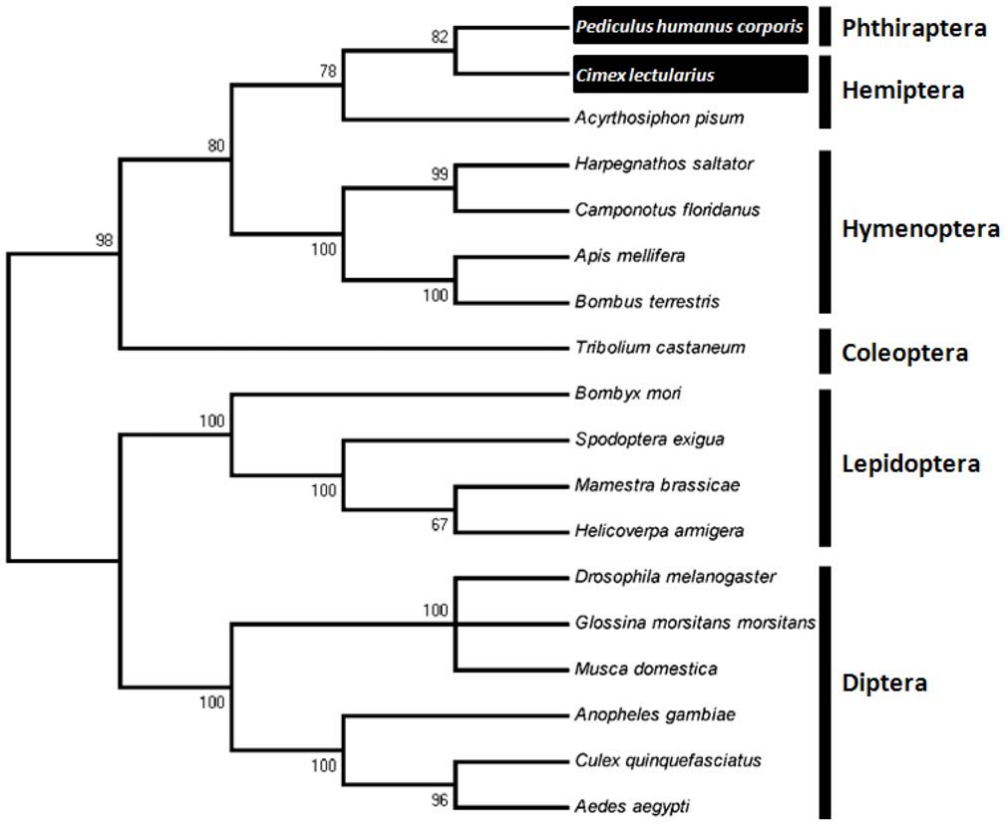
The neighbor-joining consensus tree illustrates the phylogenetic relationship of ClCPR with other insect CPRs. The phylogenetic tree was generated by MEGA 5 according to the amino acid sequences. All nodes have significant bootstrap support based on 2,000 replicates. All insects CPRs were clustered into six groups which was six insect orders. The CPRs from bed bug (*Cimex lectularius*) and body louse (*Pediculus humanus corporis*) which showed the closest evolutionary relationship with *ClCPR* were highlighted in black boxes.

Phylogenetic analysis was performed based on the amino acid sequences of ClCPR and 17 other insect CPR proteins (Table S1). The phylogenetic tree was generated by MEGA 5 with the neighbor-joining algorithm. As expected, insect CPRs from the same insect order were grouped together (Fig. 2). Among all the sequences, ClCPR shared the highest sequence similarity (75%) with the CPR of the body louse, *Pediculus humanus corporis* (Table S1). It was consistent with the result of phylogenetic analysis, in which ClCPR originated from a same evolutionary root with the CPR in *P. humanus corporis* with the bootstrap value of 82 (Fig. 2) even though they do not belong to the same taxonomic order.

### Bed bug reference gene selection

Since very little information is available on gene expression in the bed bug, we decided to identify a housekeeping gene for qRT-PCR analysis. Results of reference gene examination RNA isolated from whole body samples across different developmental stages and populations are shown in Table S3 and Fig. S3. Based on the two most important criteria for evaluating the stability of reference genes by the *BestKeeper* program, the stability (SD value) and the relation to the *BestKeeper* index (*r* and *P*-value), all four reference genes, *rpl11*, *rpl8*, *rps16* and *hsp70* tested are stable across developmental stages and populations tested. The *rpl8* was chosen as the reference gene to calculate relative expression levels of *ClCPR* because it showed the most stable expression among samples tested. In subsequent studies, *rpl8* expression was found to be stable across different tissues as well as dsRNA injected and control insects (data not shown). Therefore, *rp18* was used as a reference gene in all the experiments.

### Developmental, sexual, and spatial expression patterns of *ClCPR*

**Figure 3:**
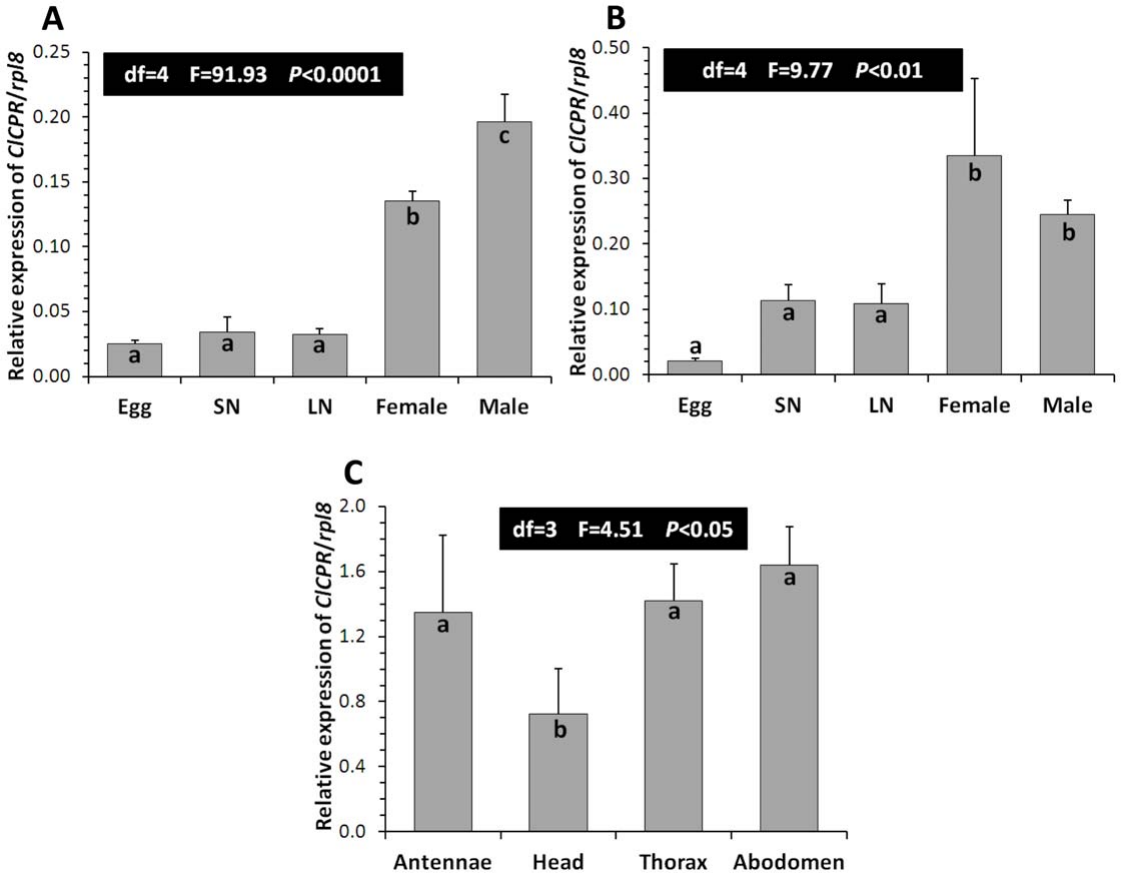
Spatial and temporal expression of *ClCPR*. Changes in mRNA levels of the *ClCPR* in CIN-1 (A) and LA-1 (B) populations. Egg; SN, small nymph (1–3 instar); LN, large nymph (4–5 instar); female and male, 1 week old. The relative mRNA levels were shown as a ratio in comparison with the levels of *rpl8* mRNA. The data shown are mean+SEM (n = 3). (C) Relative mRNA levels of the *ClCPR* in the antennae, head, thorax, and abdomen of the CIN-1. Tissues were dissected and total RNAs were isolated to quantify the *ClCPR* mRNA levels by qRT-PCR as described in Materials and Methods. Relative mRNA levels were normalized using the expression of *rpl8*. The data shown are mean+SEM (n = 4). Statistical significance of the gene expression among samples was calculated using one-way ANOVA followed by Duncan multiple mean separation techniques. There was no significant difference among relative expression within samples with the same alphabetic letter (i.e. a, b and c).

The developmental, sexual, and spatial expression of *ClCPR* gene was investigated using qRT-PCR. The mRNA levels of *ClCPR* in 1 to 5 day pooled eggs, small nymphs (1^st^ to 3^rd^ instar), large nymphs (4^th^ and 5^th^ instar), and 1 week old female and male adults in both resistant CIN-1 and susceptible LA-1 populations were quantified. Low levels of *ClCPR* mRNA were detected in eggs, small nymphs, and large nymphs in both CIN-1 and LA-1 populations (Figs. 3A and B). There were no significant differences in the *ClCPR* mRNA levels among these samples. The mRNA levels of *ClCPR* in female and male adults were significantly higher than those in the early stages of both populations (Figs. 3A and B). Moreover, the *ClCPR* mRNA was detected in the antennae, head, thorax, and abdomen isolated from female CIN-1 adults (Fig. 3C).

### Dose dependent and systemic *ClCPR* knockdown

**Figure 4:**
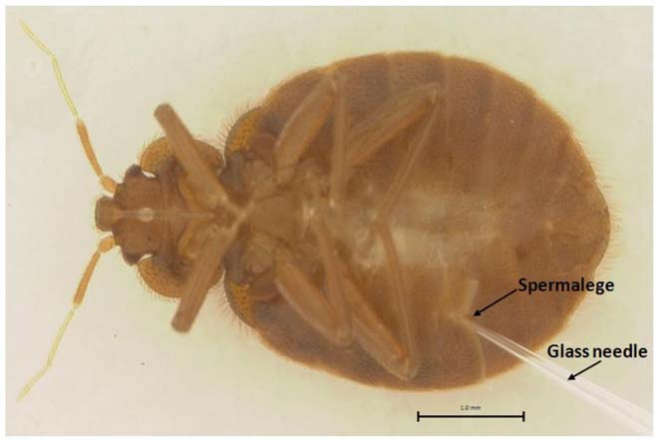
Bed bug dsRNA injection by mimicking traumatic insemination. Female bed bug showing the site of dsRNA injection via the spermalege using a sterilized glass needle.

The bed bugs are unusual in being a gonochorist taxon with obligate traumatic insemination. During the process of copulation, the male pierces the female's abdominal wall and transfers sperm into her haemocoel [2]. However, the female bed bugs evolved a unique anatomical organ, the spermalege, into which the male punctures the female with a needle-like paramere. Research suggested that the evolution of spermalege resulted from selection to defense against mating-associated pathogens [45]. Preliminary studies showed that injection of dsRNA through spermalege caused lower mortality compared to the injections at the other sites in the abdomen. Therefore, the dsRNAs were routinely injected through the spermalege into the body of female bed bugs (Fig. 4). Bed bugs injected with *malE* or *ClCPR* dsRNA suffered similar rate of mortality within 5 days after injection (most of them died in the first one or two days) (Fig. S4). There was no other obvious negative effects caused by injecting *ClCPR* dsRNA observed during the 6-day experimental period (including 5 days after injection and 24 h for bioassay).

**Figure 5:**
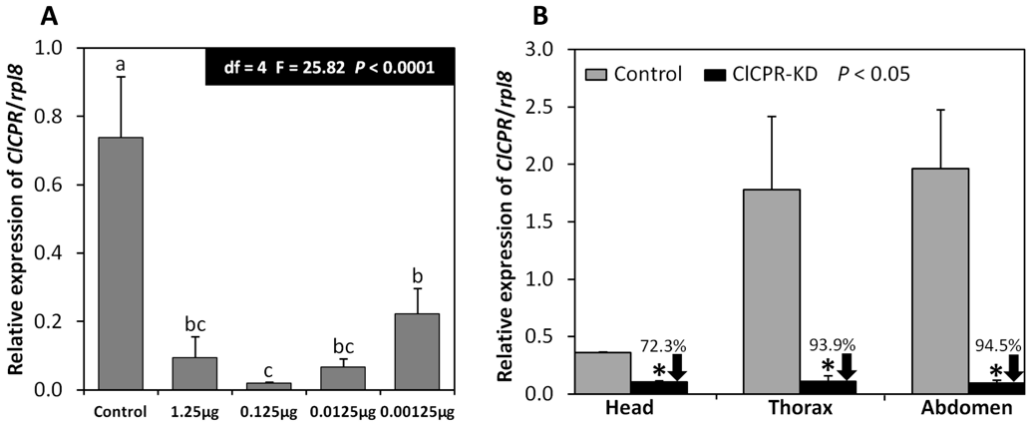
Relative *ClCPR* mRNA levels in control (*malE* dsRNA) and *ClCPR* dsRNA injected bed bugs. (A) The *ClCPR* mRNA levels were quantified by qRT-PCR at 5 days after dsRNA injection in control (*malE* dsRNA) and *ClCPR* dsRNA treated bed bugs with different doses of dsRNA. (B).Relative *ClCPR* mRNA levels in different body parts in control (*malE* dsRNA) and *ClCPR* dsRNA injected bed bugs. The relative *ClCPR* mRNA levels are shown as a ratio in comparison with the levels of *rpl8* mRNA. The data shown are mean+SEM (n = 3).

A preliminary study showed that 1.25 µg of *ClCPR* dsRNA was sufficient to silence *ClCPR* gene in each individual bed bug. In order to identify the most effective dose for silencing the *ClCPR* gene, serial 10-fold dilutions of *ClCPR* dsRNA were injected and the *ClCPR* mRNA levels were quantified using qRT-PCR and total RNA isolated at 5 days after injection of dsRNA. As shown in Figure 5A, 0.125 µg/individual of *ClCPR* dsRNA was the most effective dose to suppress the expression of *ClCPR* gene in CIN-1 population. Subsequently, we detected *ClCPR* knockdown efficiency in different body parts, including head, thorax, and abdomen. RNAs extracted from these body parts of both control (injected with dsRNA of *malE*, a bacterial gene) and *ClCPR* dsRNA treated bed bugs were subjected to qRT-PCR analysis. The *ClCPR* gene was successfully suppressed in all body parts tested, indicating that the RNAi effect in bed bugs is systemic (Fig. 5B).

### *ClCPR* knockdown increases CIN-1 and NY-1 sensitivity to deltamethrin

**Figure 6:**
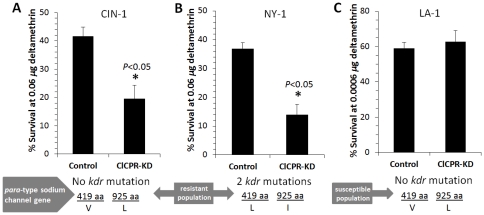
Knockdown in the expression of *ClCPR* reduced the resistance to deltamethrin. (A) The percent survival of dsRNA treated CIN-1 (deltamethrin resistant population without para-type sodium channel gene mutation at 419 aa and 925 aa) bed bugs at 0.06 µg deltamethrin 5 days after dsRNA injection. The mortality was recorded after 24 h exposure to deltamethrin (3 replicates, 50–60 individuals for each replicate). (B) The % survival of dsRNA treated NY-1 (deltamethrin resistant population with 2 para-type sodium channel gene mutations at 419 aa and 925 aa) bed bugs at 0.06 µg deltamethrin 5 days after dsRNA injection. The mortality was recorded after 24 h exposure to deltamethrin (3 replicates, 38–40 individuals for each replicate). (C) The % survival of dsRNA treated LA-1 (deltamethrin susceptible population without para-type sodium channel gene mutation at 419 aa and 925 aa) bed bugs at 0.0006 µg deltamethrin 5 days after dsRNA injection. The mortality was recorded after 24 h exposure to deltamethrin (3 replicates, 60 individuals for each replicate). The differences between control and CPR-KD in three bed bug populations were analyzed by Student's *t*-test.

Five days after injection of dsRNA, the survived bed bugs were exposed to deltamethrin through topical application. The percent survival was recorded after 24 h exposure to deltamethrin. The *ClCPR* knockdown in deltamethrin resistant populations CIN-1 (no *kdr* mutation) and NY-1 (two *kdr* mutations) bed bugs showed a consistent increase in susceptibility to deltamethrin compared with control bed bugs (Figs. 6A and 6B). In contrast, there was no significant difference in the susceptibility to deltamethrin between *ClCPR* knockdown and control in insecticide susceptible LA-1 bed bugs (Fig. 6C).

## Discussion

### Overview

The main goal of this study is to characterize NADPH-Cytochrome P450 reductase from the bed bug and investigate whether the P450-mediated metabolic detoxification plays any role in the deltamethrin resistance of bed bugs. To achieve the goal, the *ClCPR* gene was isolated from a deltamethrin resistant population (CIN-1) with a combined PCR strategy (Fig. S1). Three conserved binding domains, a FAD binding motif, and the catalytic residues as well as the critical residues involved in FMN, FAD and NADP binding were identified (Fig. 1). The spatial configuration and the putative functions of these conserved domains were analyzed by predicting a 3-D model of ClCPR (Fig. 1B). The ClCPR was predicted anchoring on the membrane of endoplasmic reticulum by a 21 amino acids transmembrane region (Figs. 1A and S2). The phylogenetic analysis showed that ClCPR had the shortest genetic distance to the CPR from body louse (Fig. 2). The *ClCPR* gene was ubiquitously expressed in all tissues tested (Fig. 3A and B) but showed an increase in expression as immature stages develop into adults (Fig. 3A). With mimicking the traumatic insemination of bed bugs, dsRNA of *ClCPR* was injected into the bed bug and successfully suppressed the expression of the gene coding for ClCPR throughout the body (Figs. 4 and 5). Similar to the previous report from *An. gambiae* [26], when the *ClCPR* was suppressed through RNAi in deltamethrin resistant bed bug populations, the susceptibility of these bed bugs to deltamethrin was significantly enhanced. In this study, the susceptibility enhancement was observed in populations containing *kdr* mutation (NY-1, Fig. 6B) as well as no-*kdr* mutations (CIN-1, Fig. 6A), but not in the susceptible population (LA-1, Fig. 6C), suggesting P450-mediated metabolic detoxification may serve as one of the resistance mechanisms employed by bed bugs.

### *ClCPR* gene discovery and analysis

As an obligatory electron donor, CPR transfers electrons from NADPH to various cytochrome P450s that play central roles in detoxification of xenobiotics [41]. Consequently, identification and characterization of CPR from insects will help to determine whether or not Cytochrome P450s are involved in response of insects to specific insecticides and other xenobiotics [26], [28]. The primary structures of CPRs are highly conserved across diverse taxa, indicating the functional importance of this enzyme throughout the course of evolution [46]. The alignment of ClCPR with other CPRs showed that ClCPR shares 64–75% amino acid identity with other insect CPRs, and 54% with human (*Homo sapiens*), rat (*Rattus norvegicus*) and mouse (*Mus musculus*) CPRs. The structure analysis of ClCPR demonstrated that ClCPR has four functional domains (Fig. 1). The hydrophobic N-terminal membrane anchor of ClCPR is essential for its function in the P450 catalytic cycle. It serves to anchor the protein molecule to the endoplasmic reticulum which ensures proper spatial interaction for electron transfer between the ClCPR and cytochrome P450s [41], [46]. Without the hydrophobic anchor, ClCPR is incapable of transferring electrons to cytochrome P450s. ClCPR also has three distinct protein binding domains. At the N-terminal, FMN binding domain consists of two FMN binding sites, FMN1 and FMN2 (Figs. 1A and C). FMN1 and FMN2 binding sites were well conserved among all insect CPRs indicating they play critical roles in the interaction with cytochrome P450s (Fig. 1C). For example, mutation of residues corresponding to Asp 211 and Thr 93 in *C. lectularius* to alanine in the yeast CPR resulted in complete loss of functional activity toward CYP51 [42].

The phylogenetic analysis showed that *ClCPR* had the closest evolutionary relationship with the CPR from the body louse. Although the bed bug and the body louse do not belong to the same taxonomic order and occupy different living habitats, these two species are both obligatory hematophagous (bloodsucking) insects which maintain close association with the human being throughout their life stages. Feeding on human blood implies that they share some of the same xenobiotic challenges, therefore may have evolved a similar function for their CPRs.

### Tissue distribution of *ClCPR*

The primary tissue distribution of *CPR* is associated with the potential localization of P450 activities, which in turn reflect the prospective function of P450s. For example, the *D. melanogaster CPR* was expressed more abundantly in embryos and antennae as compared to adult heads, adult bodies and larvae [23] indicating the functions of *D. melanogaster* P450s in embryonic development and odorant clearance. The cabbage armyworm, *M. brassicae*, *CPR* was predominantly expressed in all tissues tested including male and female antenna, male brain, proboscis, thorax, abdomen, and legs and female ovipositors. The cellular localization of *CPR* and two P450s within antennae suggest the potential importance of cytochrome metabolism in the olfactory sensilla of the cabbage armyworm [25]. *An. gambiae CPR* was mainly localized in the antennae, mid-gut epithelia and oenocytes which are considered to be a major spot for heme biosynthesis in insects [26]. These observations suggest potential roles of *A. gambiae* P450s in odorant metabolism, insecticides metabolism, and regulating heme homeostasis. All insects are living in their environments surrounded by various chemicals, volatile or nonvolatile, natural or anthropic, useful or harmful. The pores located on the antenna allow the entrance of massive odor molecules which potentially trigger the olfactory signaling transduction in the olfactory sensillum and subsequently are metabolized by odorant-degrading enzymes (including Cytochrome P450s) in the neuroepithelium or the brain [25]. In this study, *ClCPR* was detected to be ubiquitously distributed throughout the body, including antenna, head, thorax, and abdomen (Fig. 3C). The significant expression of *ClCPR* in the antenna demonstrates the potentially functional importance of bed bug P450s in the odorant clearance and/or xenobiotics metabolism which might facilitate the host identification and localization in bed bugs. On the other hand, the broad presence of *ClCPR* in the head and abdomen may imply the roles of bed bug P450s in xenobiotics metabolism and/or endogenous compound biosynthesis.

### Inactivation of *ClCPR*

Inactivation of *CPR* as a whole has been proposed to cause multiple developmental defects and embryonic lethality in mouse [47], [48] and increase the permethrin susceptibility in *An gambiae* [26]. In this study, RNAi was tested in the bed bug, and the RNAi effect was found to be dsRNA dose-dependent and systemic. When the *ClCPR* was suppressed through RNAi in deltamethrin resistant populations, the susceptibility of these bed bugs to deltamethrin was significantly enhanced. The susceptibility enhancement was not only observed in non-*kdr* mutation population (CIN-1, Fig. 6A) but also observed in the population that contains *kdr* mutation (NY-1, Fig. 6B) where decreased target-site sensitivity of voltage-gated sodium channels was suggested as one of the mechanisms of pyrethroid resistance. These data suggest that multiple resistance mechanisms may exist in NY-1 population. However, there was no significant difference in the susceptibility to deltamethrin between *ClCPR* knockdown and control in insecticide susceptible LA-1 bed bugs (Fig. 6C). These data suggest that P450-mediated metabolic detoxification might be another mechanism responsible for deltamethrin resistance in general bed bug populations. Recent report supports our prediction by showing higher levels of P450 mRNAs in pesticide resistant populations compared to their levels in susceptible populations of *C. lectularius* [49]–[50]. Taken together, P450-mediated metabolism could be added to factors such as the target site insensitivity, behavioral and penetration mechanisms involved in the deltamethrin resistance of bed bug populations.

## Supporting Information

Figure S1 **A schematic diagram showing the strategy used to clone the full length** ***ClCPR*** **.** The top line stands for the cDNA. Other lines represent gene fragments isolated by RACE or PCR with specific PCR primer pair(s): fragment 1 (NADPHF/NADPHR), fragment 2 (ClRACER1-1/UPM, ClRACER2-1/NUP), fragment 3 (ClRACEF1-1/UPM, ClRACEF2-1/NUP), fragment 4 (ClRACEF3/UPM, ClRACEF4/NUP), and fragment 5 (ClCPRF/ClCPRR).
(TIF)

Figure S2 **Transmembrane helix (A and B) and hydrophobicity (C and D) of ClCPR prediction.** The total 679 amino acids (A) and 140 N-terminal amino acids (B) were submitted into TMHMM Server 2.0 in turn. A 21-amino acid transmembrane region was predicted and highlighted in red. The total 679 amino acids (C) and 140 N-terminal amino acids (D) were also submitted into the on line molecular tool, Protein Hydrophobicity Plots. The hydrophobicity profiles of ClCPR were delineated using Kyte-Doolittle scale. Regions with values above 0 are hydrophobic in character.
(TIF)

Figure S3 **Selection of a reference gene.** Stable expression of four reference genes, *rpl11*, *rpl8*, *rps16* and *hsp70* are shown across 8 RNA samples isolated from different developmental stages: eggs (1,5), small nymphs (1–3 instar) (2,6), large nymphs (4–5 instar) (3,7), and 1 week old female and male (4,8) adults in CIN-1 (1–4) and LA-1 (5–8). Products obtained after 40 cycles of PCR amplification under conditions described in the Materials and Methods section were resolved on an agarose gel and the gel was stained with ethedium bromide.
(TIF)

Figure S4 **The average mortality of bed bugs at 5 days after dsRNA injection.** No significant difference was observed in the mortalities between *ClCPR* dsRNA and *malE* dsRNA (control) injected bed bugs (*n* = 7; Student's *t*-test, *P* = 0.49).
(TIF)

Table S1 **ClCPR homologues in insects.**
(DOCX)

Table S2 **Primers used for cloning, RACE, qRT-PCR, RNAi, and housekeeping gene analysis.**
(DOCX)

Table S3 **Statistical analyses of four candidate reference genes based on their threshold cycle (C_T_) value.**
(DOCX)
