# Supplementary figures and images for: RNA Interference of NADPH-Cytochrome P450 Reductase Results in Reduced Insecticide Resistance in the Bed Bug, Cimex lectularius
Source: PLoS One. 2012 Feb 7;7(2):e31037. doi: 10.1371/journal.pone.0031037 (PMC3274526; doi:10.1371/journal.pone.0031037)

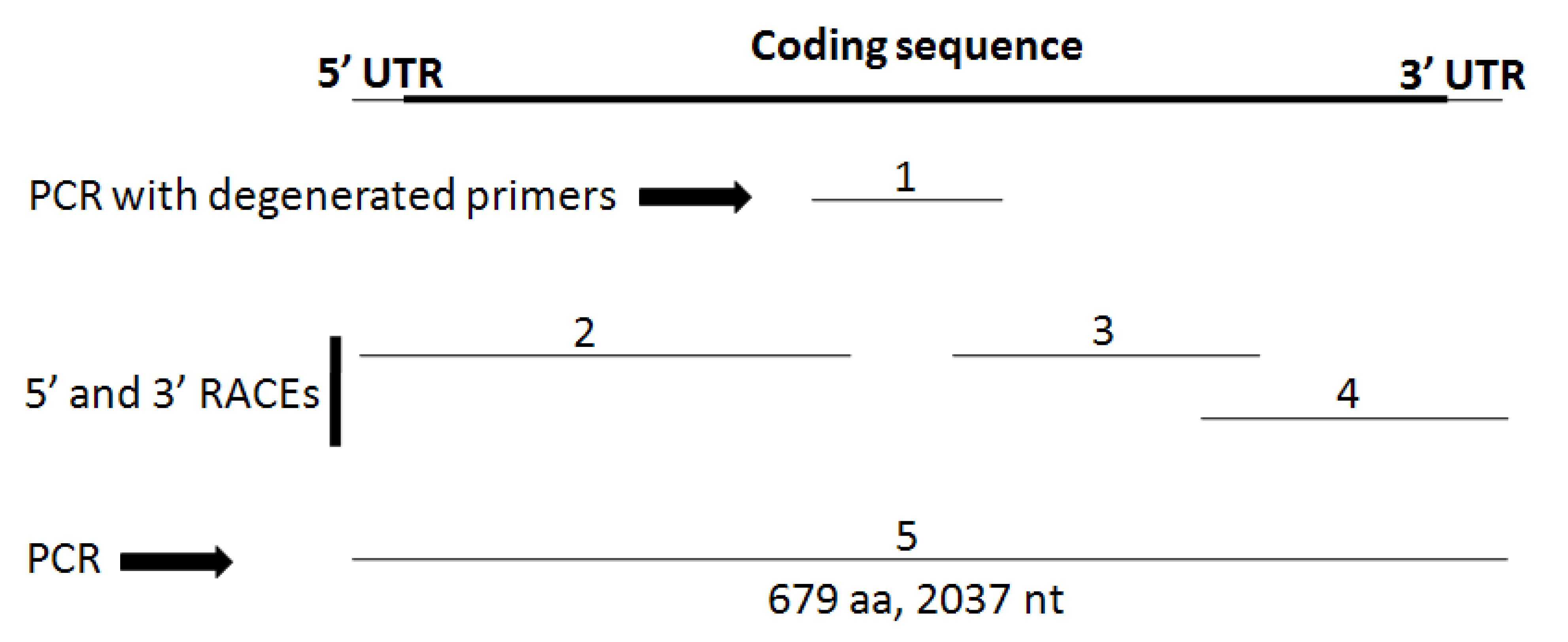

Supplement: Figure S1 — A schematic diagram showing the strategy used to clone the full length ClCPR . The top line stands for the cDNA. Other lines represent gene fragments isolated by RACE or PCR with specific PCR primer pair(s): fragment 1 (NADPHF/NADPHR), fragment 2 (ClRACER1-1/UPM, ClRACER2-1/NUP), fragment 3 (ClRACEF1-1/UPM, ClRACEF2-1/NUP), fragment 4 (ClRACEF3/UPM, ClRACEF4/NUP), and fragment 5 (ClCPRF/ClCPRR). (TIF) [file pone.0031037.s001.tif]

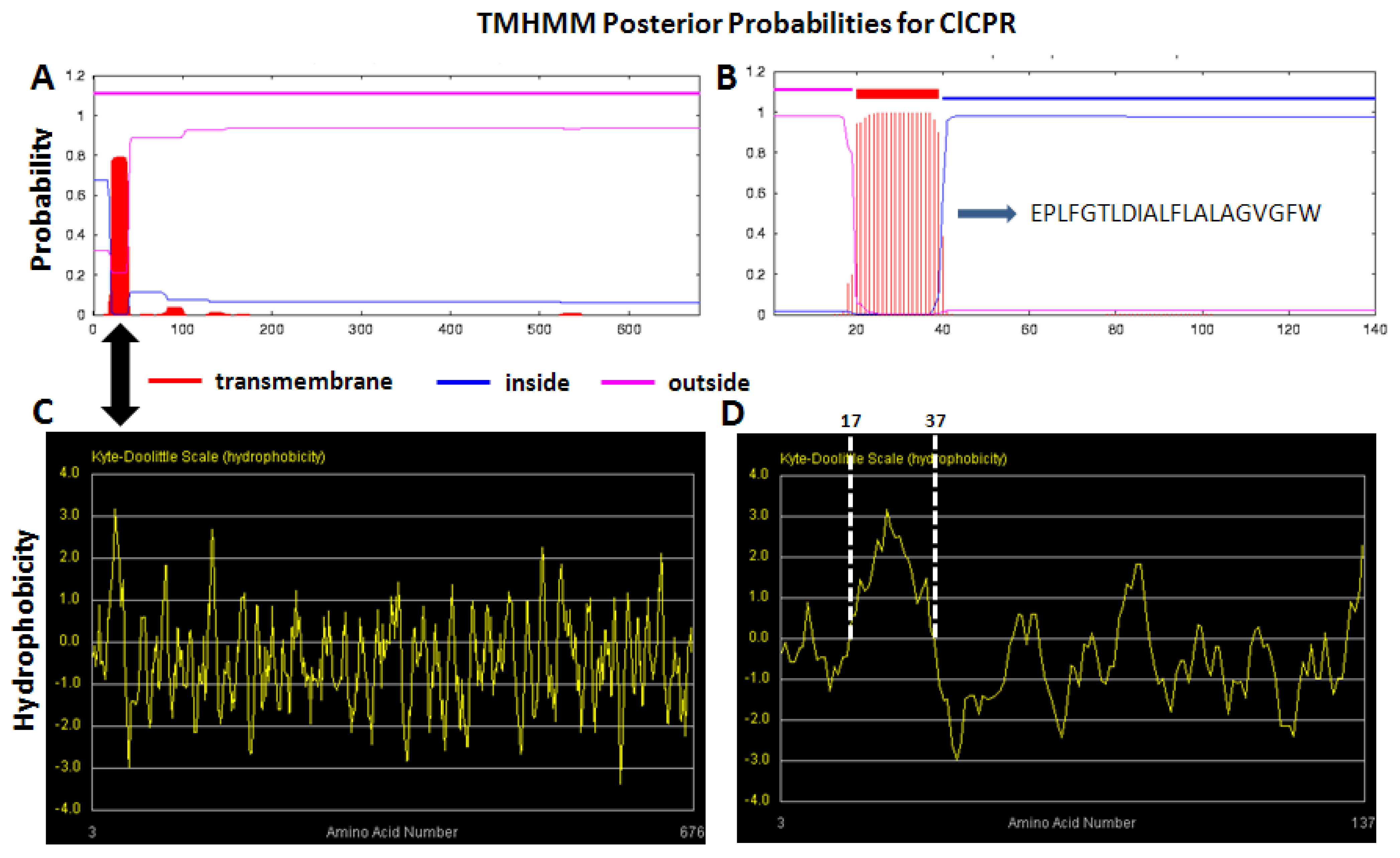

Supplement: Figure S2 — Transmembrane helix (A and B) and hydrophobicity (C and D) of ClCPR prediction. The total 679 amino acids (A) and 140 N-terminal amino acids (B) were submitted into TMHMM Server 2.0 in turn. A 21-amino acid transmembrane region was predicted and highlighted in red. The total 679 amino acids (C) and 140 N-terminal amino acids (D) were also submitted into the on line molecular tool, Protein Hydrophobicity Plots. The hydrophobicity profiles of ClCPR were delineated using Kyte-Doolittle scale. Regions with values above 0 are hydrophobic in character. (TIF) [file pone.0031037.s002.tif]

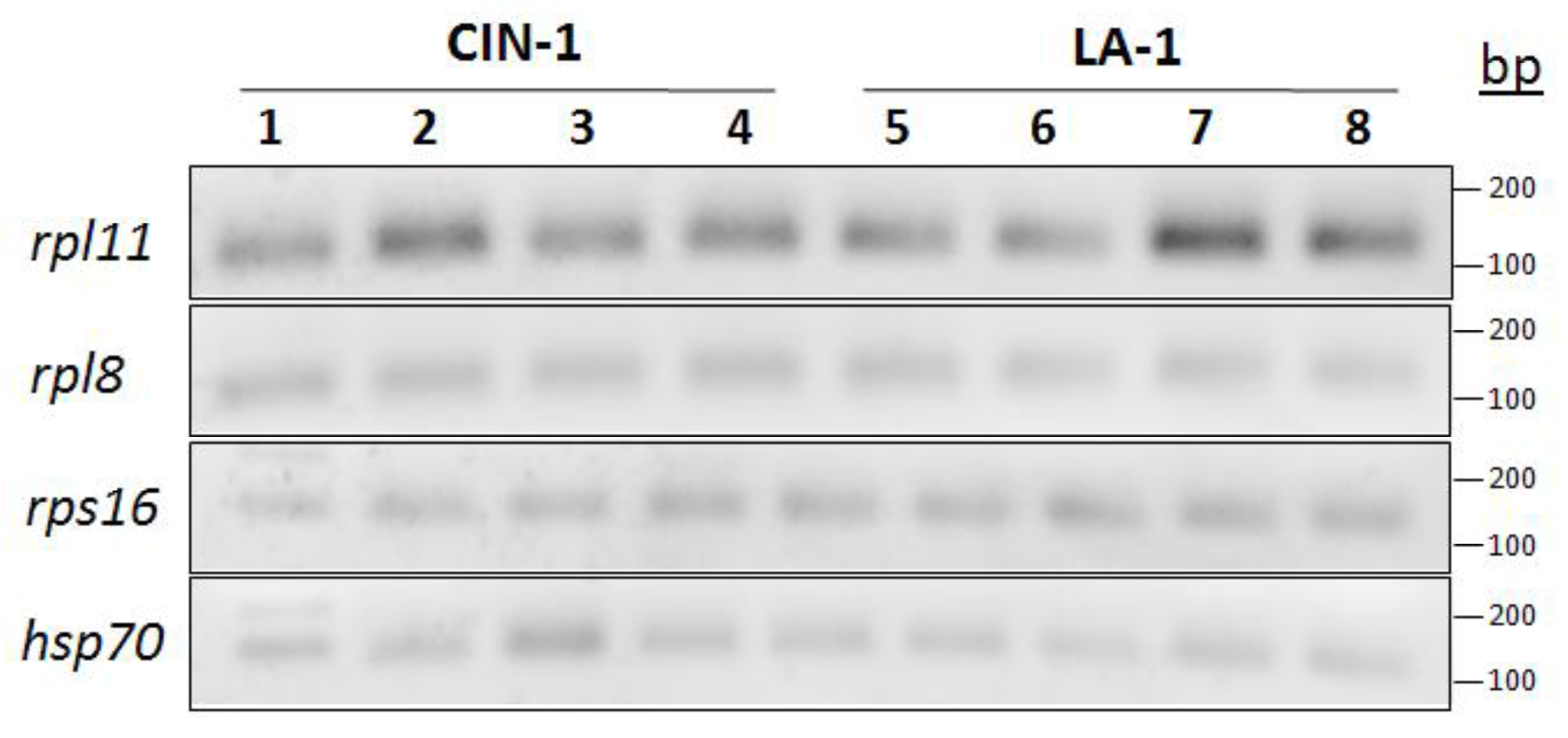

Supplement: Figure S3 — Selection of a reference gene. Stable expression of four reference genes, rpl11, rpl8, rps16 and hsp70 are shown across 8 RNA samples isolated from different developmental stages: eggs (1,5), small nymphs (1–3 instar) (2,6), large nymphs (4–5 instar) (3,7), and 1 week old female and male (4,8) adults in CIN-1 (1–4) and LA-1 (5–8). Products obtained after 40 cycles of PCR amplification under conditions described in the Materials and Methods section were resolved on an agarose gel and the gel was stained with ethedium bromide. (TIF) [file pone.0031037.s003.tif]

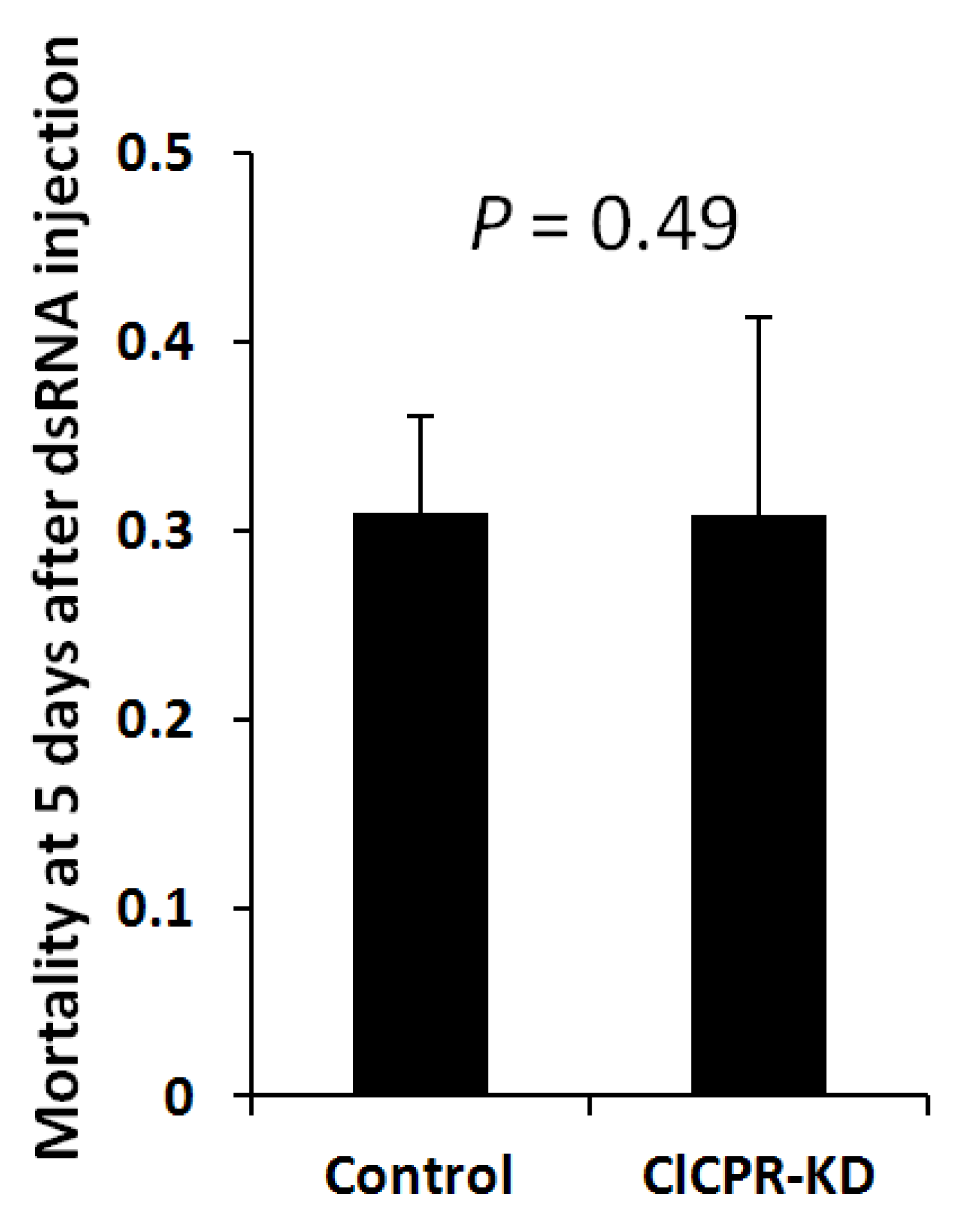

Supplement: Figure S4 — The average mortality of bed bugs at 5 days after dsRNA injection. No significant difference was observed in the mortalities between ClCPR dsRNA and malE dsRNA (control) injected bed bugs (n = 7; Student's t-test, P = 0.49). (TIF) [file pone.0031037.s004.tif]
