# Supplementary material for: RNA Interference of NADPH-Cytochrome P450 Reductase Results in Reduced Insecticide Resistance in the Bed Bug, Cimex lectularius
Source: PLoS One. 2012 Feb 7;7(2):e31037. doi: 10.1371/journal.pone.0031037 (PMC3274526; doi:10.1371/journal.pone.0031037)
Supplement: Table S1 — ClCPR homologues in insects. (DOCX) [file pone.0031037.s005.docx]

Table S1. ClCPR homologues in insects

| **Insect Order** | **Insect Species** | **Accession No.** | **Length (aa)** | **Score** | **Identities** |
| --- | --- | --- | --- | --- | --- |
| [Phthiraptera](http://en.wikipedia.org/wiki/Phthiraptera) | *Pediculus humanus corporis* | XP_002423980 | 678 | 1095 | 75% |
| Hemiptera | *Cimex lectularius* | Submit | 679 | --- | 100% |
|  | *Acyrthosiphon pisum* | XP_001945312 | 681 | 1008 | 69% |
| Hymenoptera | *Harpegnathos saltator* | EFN87403 | 939 | 1018 | 72% |
|  | *Camponotus floridanus* | EFN67037 | 679 | 1029 | 70% |
|  | *Apis mellifera* | XP_001119949 | 933 | 1031 | 72% |
|  | *Bombus terrestris* | XP_003401789 | 933 | 1019 | 71% |
| Coleoptera | *Tribolium castaneum* | XP_971174 | 680 | 1035 | 73% |
| Lepidoptera | *Bombyx mori* | NP_001104834 | 687 | 910 | 64% |
|  | *Spodoptera exigua* | ADX95746 | 689 | 927 | 65% |
|  | *Mamestra brassicae* | AAR26515 | 687 | 939 | 66% |
|  | *Helicoverpa armigera* | ADK25060 | 687 | 939 | 66% |
| Diptera | *Drosophila melanogaster* | NP_477158 | 679 | 958 | 68% |
|  | *Glossina morsitans morsitans* | ADD19306 | 672 | 978 | 68% |
|  | *Musca domestica* | Q07994 | 671 | 979 | 70% |
|  | *Anopheles gambiae* | AAO24765 | 679 | 964 | 65% |
|  | *Culex quinquefasciatus* | XP_001865801 | 679 | 974 | 68% |
|  | *Aedes aegypti* | XP_001656715 | 679 | 975 | 68% |
