# Supplementary material for: RNA Interference of NADPH-Cytochrome P450 Reductase Results in Reduced Insecticide Resistance in the Bed Bug, Cimex lectularius
Source: PLoS One. 2012 Feb 7;7(2):e31037. doi: 10.1371/journal.pone.0031037 (PMC3274526; doi:10.1371/journal.pone.0031037)
Supplement: Table S2 — Primers used for cloning, RACE, qRT-PCR, RNAi, and housekeeping gene analysis. (DOCX) [file pone.0031037.s006.docx]

Table S2. Primers used for cloning, RACE, qRT-PCR, RNAi, and housekeeping gene analysis

| **Gene** | **Accession No.** | **Primer Name** | **Primer Function** | **Primer Sequence** |
| --- | --- | --- | --- | --- |
| *ClCPR* | submit | NADPHF | Cloning | 5’GARGAYGACTTYATYACDTGGAARG 3’ |
|  |  | NADPHR | Cloning | 5’TCDCCNGYNTCRTANCGCATYTT 3’ |
|  |  | ClRACER1-1 | 5’-RACE | 5’GGGAATGGAGTCTTGCCACTTCGCC 3’ |
|  |  | ClRACER2-1 | 5’-RACE | 5’CGGACACATCTTCACCGGCCCCCTC 3’ |
|  |  | ClRACEF1-1 | 3’-RACE | 5' GCGAGTATTTCGGTATTGAGGGGGCCGG 3' |
|  |  | ClRACEF2-1 | 3’-RACE | 5’GAGGGGGCCGGTGAAGATGTGTCCG 3’ |
|  |  | ClRACEF3 | 3’-RACE | 5' ACGCCGACTGGGCGAAGAAATGAAGGCG 3' |
|  |  | ClRACEF4 | 3’-RACE | 5’AGGCGTAGCGACAAGTTGGTTGGGC 3’ |
|  |  | ClCPRF | Cloning full length | 5’ATGGGTGATGCAACAGAAATG 3’ |
|  |  | ClCPRR | Cloning full length | 5’GTGTTAATTATTGCCTTTACCTTCTTG 3' |
|  |  | qClNADPHF | qRT-PCR | 5’TATGCCGCAGAATACGGACAACTC 3’ |
|  |  | qClNADPHR | qRT-PCR | 5’ACCTGCAAATTCTTCACCAGTGCC 3’ |
|  |  | dsClNADPHF-1 | RNAi | 5’TAATACGACTCACTATAGGGTGGAAGGACAAATTCTGG 3’ |
|  |  | dsClNADPHR | RNAi | 5’TAATACGACTCACTATAGGGCTCGATGTGCATACAAG 3’ |
| *rpl8* | EZ419796 | ClRpl8F | Housekeeping gene analysis | 5’AAAGGCACGGTTACATCAAAGGTG 3’ |
|  |  | ClRpl8R | Housekeeping gene analysis | 5' TAGTCTTGAACCTATAGGGGTCCC 3' |
| *rps16* | EZ419784 | ClRps16F | Housekeeping gene analysis | 5’ACAGCAGTCGCCTACTGCAAAC 3’ |
|  |  | ClRps16R | Housekeeping gene analysis | 5' TCCTGGAGTTTGTACTGGAGGACC 3' |
| *rpl11* | EZ419774 | ClRpl11F | Housekeeping gene analysis | 5’GAAGAATGTCATGCGAGATGTCAGG 3’ |
|  |  | ClRpl11R | Housekeeping gene analysis | 5' CCTTCGAGAAGACTGGCTGCTG 3' |
| *hsp70* | EZ419756 | ClHSP70F | Housekeeping gene analysis | 5’GCCTTTACAGACACGGAACGTCTG 3’ |
|  |  | ClHSP70R | Housekeeping gene analysis | 5’TCTCCCAATCAACCTTTTGGCATCG 3’ |
