# Supplementary material for: RNA Interference of NADPH-Cytochrome P450 Reductase Results in Reduced Insecticide Resistance in the Bed Bug, Cimex lectularius
Source: PLoS One. 2012 Feb 7;7(2):e31037. doi: 10.1371/journal.pone.0031037 (PMC3274526; doi:10.1371/journal.pone.0031037)
Supplement: Table S3 — Statistical analyses of four candidate reference genes based on their threshold cycle (CT) value. (DOCX) [file pone.0031037.s007.docx]

Table S3. Statistical analyses of four candidate reference genes based on their threshold cycle (C_T_) value

| *BestKeeper analyses of candidate reference genes (n=4)* | | | | | |
| --- | --- | --- | --- | --- | --- |
| Parameter | *rpl11* | *rpl8* | *rps16* | *hsp70* | *BestKeeper* |
| N | 64 | 64 | 64 | 64 | 64 |
| GM [C_T_] | 16.32 | 16.35 | 16.51 | 15.81 | 18.53 |
| AM [C_T_] | 16.33 | 16.37 | 16.52 | 15.84 | 18.56 |
| Min [C_T_] | 14.88 | 14.85 | 14.78 | 14.29 | 16.72 |
| Max [C_T_] | 17.73 | 18.15 | 18.16 | 18.36 | 21.33 |
| SD [± C_T_] | 0.51 | 0.52 | 0.56 | 0.83 | 0.86 |
| CV [% C_T_] | 3.14 | 3.19 | 3.37 | 5.21 | 4.63 |
| *Pair-wise correlation analyses* | | | | | |
| *BestKeeper* vs | *rpl11* | *rpl8* | *rps16* | *HSP70* |  |
| Coeff. of corr. [*r*] | 0.983 | 0.996 | 0.985 | 0.984 |  |
| Coeff. of det. [*r^2^*] | 0.966 | 0.992 | 0.970 | 0.968 |  |
| *P*-value | 0.001 | 0.001 | 0.001 | 0.001 |  |

Abbreviations for listed parameters: n: number of candidate reference genes; N: sample size for each candidate reference genes as well as the *BestKeeper*; GM [C_T_]: geometric means of the threshold cycle (C_T_); AM [C_T_]: the arithmetic mean of C_T_; Min [C_T_] and Max [C_T_]: the extreme values of C_T_; SD [± C_T_]: the standard deviation of the C_T_; CV [% C_T_]: the coefficient of variance expressed as a percentage at the C_T_ level; The correlation between each candidate reference gene and *BestKeeper* index is calculated by the Pearson correlation coefficient [*r*], coefficient of determination [*r^2^*], and the *P*-value.
